# Estimating the economic burden attributable to online only child sexual abuse offenders: implications for police strategy

**DOI:** 10.3389/fpsyg.2023.1285132

**Published:** 2024-01-04

**Authors:** Susan Giles, Laurence Alison, Michael Humann, Ricardo Tejeiro, Hayley Rhodes

**Affiliations:** University of Liverpool, Liverpool, United Kingdom

**Keywords:** child sexual abuse, online child grooming, online child protection, economic impact, prioritization, policing, risk assesment

## Abstract

Evidence is beginning to emerge of the serious negative effects online only child sexual abuse (OOCSA) can have on victims. Establishing the scale and nature of the problem could assist police in prioritizing suspects. In study 1, scoping review identified eleven studies that examined OOCSA's impact on victims. Five themes emerged from narrative review; *definitional issues, a new normal, OOCSA grooming processes, comparisons with offline CSA, mechanisms between OOCSA and harm*. In study 2, OOCSA national prevalence was estimated by applying 2.9% rate of OOCSA observed from original police data to a lower bound (“sexual communication with a child” crimes recorded by the police), middle (scaling up to estimate undetected offenses) and upper bound estimate of the national offender pool (self-reported sexual solicitation offenders). Recent UK Home Office figures were adapted to establish economic costs. Lifetime costs estimates attributable to OOCSA are £7.4 million (police reports), £59.6 million (including undetected offenders) and £1.4 billion (national prevalence estimates). Over 75% of this is non-financial costs borne by victims in terms of emotional harm and lost output. Government bears around 20% of the cost burden, mostly non-financial costs for police forces. Findings are discussed in relation to evidence-led recommendations for prioritization and wider police actions that can be taken to avoid the considerable economic and social burden associated with OOCSA offenses.

## Introduction

An estimated 680,000 to 830,000 UK based adult offenders pose varying degrees of sexual risk to children (NCA, 2023). Of which, an earlier estimate by the Home Office suggests 80,000 pose a risk of online harm. Whilst contact offenders remain a top priority for law enforcement, contributing an economic cost burden of £10.1 billion (Radakin et al., 2021), the online offenders should be seen as an increasing priority due to the harm caused to large numbers of victims. There has been recognition of the serious negative effects online only child sexual abuse (OOCSA), including grooming and related online sexual abuse, can have on victims (Hamilton-Giachritsis et al., 2017). Specifically, there is some evidence that the emotional, psychological and social effects upon victims may be similar to that caused by contact offending (Whittle et al., 2013; Hamilton-Giachritsis et al., 2017). Arguably, from a policing perspective, these levels of harm should be key safeguarding considerations during suspect prioritization decisions. Kloess et al. (2019) suggest further, that harms being equivalent, OOCSA might carry the same prison sentence as offline CSA.

Investigators wish to respond appropriately, in terms of resource allocation, to the most concerning cases. IICSA (2020) sheds light on the increasing scale of online grooming and Fisher et al. (2017) note that online-facilitated CSA impacts on long-term health and wider outcomes for victims, representing a key evidence gap. However, there is less systematic knowledge about scale of offending that is initiated and stays online nor how it relates to victim harm. Drawing upon a scoping review and economic approach, the current research aims to explore the scale of the problem. An economic framework is developed and applied to population estimates of OOCSA offenders. The use of economic arguments can be used to justify additional resources needed to develop and deliver an effective policing response.

Understanding the problem scale is problematic due to definitional issues. There are a wide variety of terms used to describe relevant forms of offending, however few distinct definitions of online offending exist. A working definition was proposed for the current research in consultation with a group of practitioners representing a small number of UK police forces. This aligns closely with the emerging, empirically-based typology “child sexual abuse through online interaction” (CECSA, 2020). The current working definition refers to *sexually abusive and exploitative behavior toward children online. Such behaviors can cause high levels of emotional, psychological and social distress among victims, where contact offending is generally absent. The term encompasses a wide range of behaviors in which children are persuaded, pressed or coerced into engaging in online sexual behaviors. These behaviors include: causing or inciting a child to engage in sexual activity; grooming behaviors; sexual communication; producing first generation indecent images of children (IIOC) and sextortion*.

A clear understanding of the extent to which such offending harms victims is lacking. A critical literature review would aid in the assessment of both the quantity and quality of evidence exploring OOCSA related harms. Further, it would develop an understanding of online specific aspects of criminal behavior that contribute to harm. Hanson (2017) warns that the distinction between OOCSA and offline offenses is a false dichotomy as there is too much cross-over between crimes. However, researchers and practitioners need to understand how online offending achieves its aversive harm mechanisms. Such online mechanisms might be considered behavioral targets for suspect prioritization in cases where victims are offended against online only or in combination with offline offenses.

Suspect prioritization processes exist in the United Kingdom (UK) through the use of the Kent Internet Risk Assessment Tool (KIRAT: Long et al., 2016) to prioritize indecent image of children (IIOC) offenders who demonstrate risk markers for contact offending. However, if OOCSA offenders contribute to similar outcomes for victims, investigators might consider KIRAT type approaches that prioritize OOCSA offenders even without contact offending markers. For example, KIRAT currently uses grooming and sexual communication online or offline as risk factors for contact abuse (Long et al., 2016). If OOCSA offenders are not required to make contact to harm victims, grooming and sexual communication online might form a more prominent basis for an OOCSA related tool. Typically high risk markers, such as access to children, may be less critical whilst other behaviors, as yet unidentified, might have more prominence in OOCSA prioritization. A scoping review will help researchers and practitioners understand the supporting evidence for prioritization decisions.

As recommendations may impact upon the allocation of police resources it is imperative that police forces understand the scale of the problem, in terms of the number of offenses however, quantifying OOCSA prevalence proves challenging. The offending landscape has changed in recent years and recording procedures do not reliably discern OOCSA. Prevalence estimates need to reflect the police forces' situation now. There are problems with using offender typologies as the basis for prevalence estimates. There is too much overlap between typologies and online behaviors cannot reliably distinguish between typologies and offenders (e.g., Broome et al., 2018). The CSA Centre's typology is promising. However, no prevalence rates have yet been provided and the CSA Center acknowledges that offenders can belong to more than one typology.

Alternative data sources have their limitations. For example, there are several reasons for excluding victim surveys. Children and young person surveys typically ask about sexual requests or online grooming. Taking prevalence at the level of individual behaviors could get us closer to OOCSA behaviors. Karayianni et al. (2017), for example, report that 5.9% of their Cypriot sample of 15–25 year olds had been asked by an adult to record themselves or see them live via the internet for the purpose of sexual arousal. Whilst the majority of participants experienced sexual abuse between the ages of 12 and 18 it is unclear how much of the 5.9% was experienced by children and adults in the sample. Further, it is unclear how many of the 5.9% were offended against by strangers or acquaintances. Gamez-Guadix et al. (2017) found that 1.7% of their Spanish sample of 12–15 year olds had sent images (e.g., via webcams) or videos with sexual content of themselves during the last 12 months. However, this was asked in terms of ‘voluntary' behavior rather than that elicited during sexual abuse. They also asked about cybersex, but given this was a study focused on developing factor structures, prevalence is not provided by the authors. With respect to victim survey research in general, there are few representative victim survey samples of the UK child population (NSPCC, 2019) and as Wager et al. (2018) suggest questions are not standardized. Offenders may also use a range of behaviors. Further, Livingston et al. (2014) warns against conflating risk with harm. Not all children who are approached with a sexual request online engage with offenders and are harmed, so it is imperative to look at the proportion of young people who comply with requests that require an online response. Such questions are rarely asked (e.g., Mitchell et al., 2008).

Police data is not without limits, though it might provide a starting point for prevalence. Police are required to “flag” CSA with an online element but it can be difficult to differentiate OOCSA from police records. The National Society for the Prevention of Cruelty to Children (NSPCC) draw on the Office of National Statistics and Freedom of Information requests to gather information on CSA recorded by police forces with an online element. Whilst “inciting a child to engage in sexual activity” might be a preferred indicator of OOCSA offending it is not routinely reported by the NSPCC. They do, however, provide regular updates on ‘sexual communication with a child' that might be suitable for the current study. Some might question, however, the small offending estimates driven by official figures given that CSA is one of the most under-reported offenses.

Academics and practitioners have explored rates of undetected CSA offending. The Children's Commissioner for England (2015) estimated that only 1 in 8 CSA offenses come to attention of statutory authorities. The literature explores many barriers to disclosure and many children do not disclose their abuse until they are adults. Research has examined undetected offenses from offenders' perspectives also. DeLisi et al. (2016), for example, found that 69% of offenders self-reported undetected contact sexual offenses during polygraph examination, of which 34 had no official record and offended against 148 victims. Drury et al. (2020) also found that 59% of IIOC offenders with no official record self-report contact offenses. Whilst adding some reservation to whether an offender can ever truly be classed as online only, it does help provide some assurance around “scaling up” from police reports to estimate a more realistic number of OOCSA offenders than those gained from official figures only.

Alternative strategies might include population surveys. Giles and Alison (2021), for example, utilized representative surveys of community males to estimate the prevalence of individuals with pedophilia and hebephilia who pose a risk of contact offending. Similarly, Wager et al. (2018) drew upon two population surveys (Bergen et al., 2015; Schultz et al., 2016) to conclude that the proportion of adults holding sexualised conversations with a child is “unlikely” to be “below the lowest estimate of 1 in 10 adults” (Wager et al., 2018; IICSA, 2020). As with the victim surveys above, caution is advised. There are no representative samples of UK adults. Bergen et al. (2015) and Schultz et al. (2016) used European non-representative online samples and both targeted pedophile forums. Schultz et al. (2016) however, report findings from two sample groups separately. The prevalence of online sexual solicitation from general website respondents was still 1 in 10 (adolescents) and 1 in 100 (child). Focusing upon sexual solicitation of minors who were strangers within the last year, they found that offenders (general website and pedophile forum respondents) tend to be young (average 24.5 years and 28.5 years for child and adolescent soliciting offenders) and around a third are female. Further information is available from Schultz et al. (2016) adult sample. Almost a third of those who solicited minors reported making contact with more than 20 individual minors, 57% had interactions that lasted several days. Nearly half (47.5%) reported a sexual outcome, including 3.5% that had engaged in cybersex with an adolescent within the past year (1.1% with a child). Figures such as these provide a more objective basis for prevalence and economic considerations helping to avoid overestimating victim harm.

Once harm and prevalence have been established, an economic framework can be developed. Giles and Alison (2021) provide a starting point with economic arguments and recommendations for IIOC suspect prioritization based upon contact offending. The current research presents an opportunity to revisit the systematic review and economic approach of Giles and Alison (2021) to explore whether the economic model developed for offline victims could be applied to OOCSA victims. Clear problems emerge. For example, Giles and Alison (2021) draw upon Heeks et al. (2018; Home Office) to establish lower bound incident costs. However, in Heeks et al. (2018) costs associated with physical and emotional harm, and health services are partly based on physical injuries. These may be less present in OOCSA cases. By contrast, Letourneau et al. (2018) (United States) is used by Giles and Alison (2021) to provide lifetime costs. Letourneau et al. (2018) approach does not differentiate forms of child sexual abuse and so the economic analysis might apply to both offline and OOCSA victims. The UK Home Office has since published lifetime costs for contact CSA (Radakin et al., 2021) and since this is UK focused, these figures are drawn upon for the present research but adapted to take account of OOCSA contexts.

### Rationale for the current study

The current research aimed to produce a scoping review, from which an economic framework could be developed and applied to an estimated population of OOCSA offenders. Establishing the economic burden in this way would help demonstrate the scale and nature of the problem. This may justify the additional resources needed to develop and deliver an effective policing response. The following research objectives were set; (a) provide an assessment of OOCSA victim harm, (b) establish OOCSA prevalence figures, (c) conduct an economic analysis of prevalence figures and (d) provide recommendations for investigative strategies based on these findings.

## Study 1: evidence base related to OOCSA victim harm

### Materials and methods

A scoping review was preferred to a systematic review (see Munn et al., 2018) as the literature is at a developmental stage that lacks systematic analysis of outcomes comparing OOCSA and online offenses. At this early stage, it was important to scope the body of literature, consider the types of methodologies used to explore links between OOCSA offending and harm, as well as consider the types of OOCSA behaviors that might form a useful basis for prioritization decisions. A range of resources were utilized to improve review quality (Moher et al., 2010; Varker et al., 2015; Mokkink et al., 2017; Munn et al., 2018; Prinsen et al., 2018; Terwee et al., 2018). A search strategy was developed setting review aims and eligibility criteria. A PICO framework was reviewed by the academic research team and police practitioners. Material was considered for inclusion if it provided empirical data from a study examining some aspect of OOCSA related victim harm. OOCSA offending was defined as online action taken by offenders to achieve sexual grooming. The preferred behavioral definition of grooming being that of Elliot (2015); “*a series of explicit or implicit goal-directed behaviors that together share the intention of preparing a target individual, where his or her compliance and/or submission is advantageous and/or necessary for the specific purpose of achieving an unlawful or exploitative goal”* (pg 87). An exploitative goal in online contexts could include (although not exclusively) sexual communication, producing or sharing IIOC or sexual abuse via webcam. Harm outcomes were defined as adverse effects reported by victims that could include health, physical, psychological, emotional, family or treatment effects. This could involve qualitative or quantitative data, exploring OOCSA victims' experiences only or in comparison with offline victims. Material was excluded if empirical data was not provided.

### Search strategy

Search terms and BOOLEAN phrases were developed from the PICO framework (“cost” OR “value” OR “economic” or “willingness to pay” OR “lost output” OR “productivity” OR “crime harm index” OR “quality life years” OR “outcomes”) AND (“victim” OR “health” OR “physical” OR “emotional” OR “family” OR “treatment” OR “victim services” OR “health services” OR “security” OR “court” OR “police” OR “criminal justice system”) AND (“sex^*^ crime” OR “sex^*^ offender” OR “sex^*^ offender victim” OR “rape” OR “child sex^*^ abuse” OR “child porn^*^” OR “indecent image^*^ of children” OR “IIOC” or “internet sex^*^ offend^*^” OR “online sex^*^ offend” OR “contact sex offen” OR “groom^*^” OR “chat room off^*^” or “solicitation off^*^” OR “molest^*^” OR “pedo^*^” OR “paedo^*^”). Expanding upon the search strategy of Giles and Alison (2021) two additional databases were included econlit, repec along with PsychINFO, Scopus, and Web of Science (WoS). National Criminal Justice Reference System (NCJRS) and PubMed. Searches covered the period 2010–2020 and were not limited by language or publication type, or whether published or unpublished. Only publications available in full text in English were retained following the initial screening process. A request for relevant police gray literature was also sent to national policing leads.

The initial search was carried out by an external search company. Search results (9285 hits) were exported into reference management software, Endnote online. The large number of hits indicated a lack of search terms specificity. The first author applied additional criteria to identify relevant material. BOOLEAN phrases “child sexual abuse,” “child sexual abuse” AND “harm,” “child sexual abuse” AND “online” OR “internet” OR “TA-CSA” OR “CSAM” OR “images” OR “child pornography” were used to filter search results on endnote online. A short list of 560 items was identified following title screening. Eighty seven items were retained after abstract screening. Inter-rater reliability was conducted between the first and fifth authors on 20% of these 560 items to assess whether they met the inclusion criteria, resulting in 95% agreement. Differences of opinion between the reviewers were resolved through discussion, resulting in 100% agreement.

**Figure 1:**
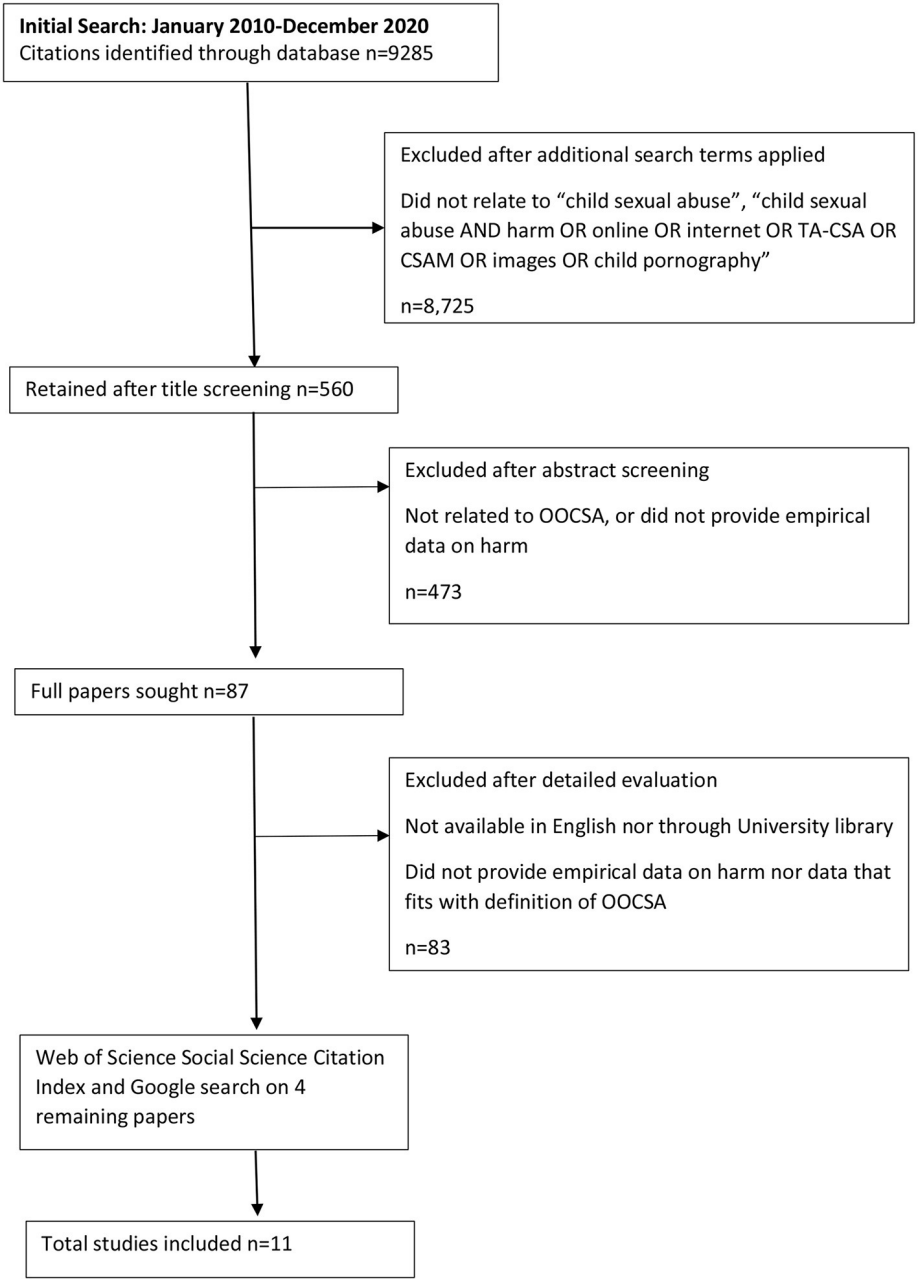
Flow diagram of study selection adapted from “Preferred items for systematic reviews and meta-analyses: the PRISMA statement,” by Moher et al. (2010).

A PRISMA flowchart is provided to help evaluate the systematic manner in which records were retained (Figure 1). Some relevant additional papers were found but were excluded because they were not available in English nor through the University of Liverpool library (e.g., Sigurjonsdottir, 2012; Bastrykin, 2017), did not provide primary data (e.g., Cooper, 2012) nor data that fits with the working definition of OOCSA (e.g., Leonard, 2010). Four studies were identified that met the study objectives and following Social Science Citation Index and Google searches a total of 11 studies were identified. A review chapter by Hanson (2017) was retained as, although it did not provide primary data, it included a synthesis of existing papers (and quotes) to forward fresh theoretical perspectives. A paper on sextortion by Wolak et al. (2018) was retained as, although it focused on sextortion, it fits with the working definition of OOCSA as some respondents were victimized as children by strangers. Included studies are summarized in Table 1.

**Table 1:** Outline of included studies RQ1 (published 2010–2020).

**References**	**Country**	**Method**	**Sample**	**Summary findings**
Beckett et al. (2019)	England and Wales	Mixed method	Surveys with 214 children and young people including focus groups with 45 children and young people. Sample includes interviews with nine young people (aged 13–20 years) who had experienced online sexual harm when aged under 18 years	9% secondary school survey participants said they had learned about online sexual harm from personal experience. Interviewees who had experienced online sexual harm placed more emphasis on the risk associated with being online and wanted others to understand the impact that it can have. Participants highlight need for education to address barriers to reporting, such as embarrassment, shame and fear of others reactions, and help around how to deal with the emotional impact of abuse Important quotes:- “*If they don't know, they won't know until it's too late. It can destroy your life*”. Sixteen years old female interview
Hamilton-Giachritsis et al. (2017, 2020a,b)	United Kingdom	Mixed method	30 questionnaires and 16 interviews with young people Childline screening questionnaire (*n =* 230 aged 17–20 years) Seven professionals responding to pilot questionnaire, 45 responding to shortened questionnaire	TA-CSA occurred in 46.4% to 62.5% of cases. TA-CSA was found to be no less impactful than offline-only sexual abuse in terms of psychological distress. Technology provided additional routes to access and control victims Online CSA victims experienced additional elements; related to control, permanence, blackmail, re-victimization and self-blame. Young people describe a range of responses from professionals, including police. Some professionals notes that, due to a lack of understanding and knowledge, online victims may be ‘blamed', seen as participating in abuse or do not see it as abuse when it is online Describe three cases, with quotes from victims, that would fit definition of HH and two of these include online CSA HH involving adult strangers
Hanson (2017)	Overview of international studies	Theoretical review	Draws on quotes from Quayle et al. (2012), Whittle et al. (2013), and Hamilton-Giachritsis et al. (2017) along with extant CSA literature to formulate theoretical perspective	Impact of offline and online CSA the same, with additional dimensions for online CSA, including increased victim participation, deception in images and image dissemination that may complicate impact and recovery. Negative trajectories (shame, blame, fear, distrust and isolation) follow abuse.
Joleby et al. (2020)	Sweden	Interviews	Seven young women (aged 17–24) with experience of online CSA before the age of 18 years	Describes three themes through thematic analysis; *From thrilling to abusive, negative effect on health and wellbeing and, a new self after the abuse*
Joleby et al. (2021)	Sweden	Analysis of court documents	Mixed method analysis of 98 legal cases of online CSA (aged 7–17 years)	Analysis of court documents demonstrated a wide range of psychological symptoms for victims of online CSA similar to those reported among victims of offline CSA (psychological distress, self-harm and suicidal behavior, sleeping problems, impaired relationships, trust issues and difficulties at school).
NSPCC (2020)	England and Wales	Transcripts of calls to NSPCC Childline and counseling sessions	Insights from transcripts organized into themes as part of briefing	Since the stay-at-home guidance was issues, Childline has seen a 11% increase in number of counseling sessions about online CSA. The NSPCC helpline saw a 60% increase in contacts from people with concerns about children experiencing online CSA Report presents multiple themes and quotes of direct relevance to the current work stream
Quayle et al. (2012)	Sweden, UK, Germany, Italy, Denmark and Russia	Interviews	27 interviews who had experienced sexual abuse offline as a result of online communication (aged 11–17 years at time of sexual abuse), 82% female	Grounded theory analysis revealed six categories; I'm missing something, Being someone who's connected, Caught in a web, Making choices, Others involvement, Closing the box and picking up the pieces
Whittle et al. (2013)	England	Interviews	Eight young people (six females and two males) aged 13–18 years old	Impact of sexual abuse not related to online/offline sexual abuse, rather correlated with level of prior vulnerability and experiences with professionals following abuse.
Wolak et al. (2018)	USA	Survey	1,550 survey respondents, 47.5% had experienced sextortion events as minors	Majority of those experiencing sextortion as minors knew the perpetrator in-person (60%), and about half did not disclose Nearly half lost relationships with friends or family members, 30% sought mental health or medical services. Some had school related problems or had to move. Minors were more likely to be pressured into producing images, threatened for more than 6 months and urged to harm themselves (compared to those over 18 years of age experiencing sextortion)

Narrative review data themes were identified using a sorting methodology. Papers were first read and summarized. They were then sorted into inductive categories that shed light on some aspect of the review objective and review question (such as OOCSA definitional or measurement problems, OOCSA related behavior, harm emerging from OOCSA, wider contexts affecting harm, and comparisons with online CSA offending). Five critical themes emerged from the narrative review.

### Results

Scoping reviews are used to assess of the density and quality of evidence on a particular issue. The lack of research in this area is in itself a key finding and many authors identify this as an emerging area of research (e.g., Beckett et al., 2019; Hamilton-Giachritsis et al., 2020a; Hanson, 2017).

#### Definitional issues

There is variation in scope and definitions used to describe OOCSA. This makes direct comparison problematic and the extent to which definitions align with definitions of OOCSA can be seen in Table 2. None of the studies dealt exclusively with OOCSA perpetrated by stranger adults. Only two studies compared the impacts of OOCSA and offline CSA [use of a control group in mixed method study by Hamilton-Giachritsis et al. (2017, 2020a) and qualitative comparisons by Whittle et al. (2013)].

**Table 2:** Definitions utilized in research with reference to OOCSA offenses (published 2010–2020).

**References**	**Definition**	**Relevance to current paper and OOCSA**
Beckett et al. (2019)	Online-facilitated child sexual abuse (OFCSA) “*online grooming and receiving sexual requests: being exposed to pornography; some sexting activities; online-facilitated child sexual exploitation (e.g., offering gifts, money or affection in return for sexual activities taking place or orchestrated online, but enacted during an offline meeting with the perpetrator or others), engaging with online images of child sexual abuse (including searching, viewing, downloading, exchanging, producing and commissioning of indecent images)”* In Beckett et al. (2019) this is revised to “online sexual harm” so as to make the language more accessible and appropriate for children and young people taking part in the research.	OFCSA is an all-encompassing definition that helps to capture the broad range of online sexual harms that can be experienced by children and young people. OFCSA can take place in both online and offline environments, including contact and non-contact abuse. Perpetrators can be known and unknown, operating alone or as part of a group, peers, older children and adults. Can include sexting. It's focus on peers, sexting and offline contact makes it broader than current definition of OOCSA
Hamilton-Giachritsis et al. (2017, 2020a,b) and Hanson (2017)	Technology-Assisted Child Sexual Abuse (TA-CSA) typology informed by victims' experiences Includes range of behaviors from peer CSA shared in victim's peer group, sexual images created consensually but shared non-consensually through to technology assisted sexual blackmail, technology assisted grooming. Hanson (2017) term preferable to online CSA as avoids ‘false dichotomy' between online and offline abuse – i.e., a child who is abused offline but who is threatened with online dissemination of images will experience the complexities of online sexual abuse identified in this review.	TA-CSA too broad a typology for narrower definition of OOCSA as it includes peer CSA and sexual images created consensually but shared non-consensually. Some aspects of TA-CSA fit well with current definition of OOCSA. For example, technology assisted sexual blackmail (TA-SB) where perpetrator engaged in online blackmail but did not attempt to take the abuse offline
Joleby et al. (2020)	Technology-Assisted Child Sexual Abuse (TA-CSA) offers a narrower working definition than typology described above “*Children have been incited to engage in online sexual activity. Such activity can include sexual chat, generating sexual photos and/or videos, performing sexual acts live via webcam, or engaging in sexually humiliating activities online*” (p. 2) Includes victims who may have perceived the offender as being a romantic partner, as well as victims who have experienced pressure and threats	Has a good overlap with the current definition of OOCSA although some of the descriptions of victim experiences might not necessarily fit within a narrow definition (e.g., peer sextortion)
Joleby et al. (2021)	Focus in this paper is on Online CSA in which the child is incited to participate actively in the abuse, for instance by producing nude or semi-nude pictures of performing sexual acts live in front on a webcam	Maps directly onto current definition of OOCSA
NSPCC (2020)	Report focusses on NSPCC helpline contacts and Childline counseling sessions where there were concerns about people specifically using the internet to target children for sexual abuse (e.g., grooming or sexual harassment)	Ties in well with current definition of OOCSA but does include peers as well as adult perpetrators
Quayle et al. (2012)	All interviewees had suffered offline sexual abuse as a result of online communication	Since each child was abused offline this focus could be seen as too broad for current definition of OOCSA but retained as contact was initiated online
Whittle et al. (2013)	Online initiated sexual abuse, including cases of online sexual grooming that lead to online or offline child sexual abuse	Tie in well with current definition of OOCSA but does also include contact offenses
Wolak et al. (2018)	Sextortion definition – Someone threatened to show a sexual image of you to another person or post it online to make you do something	Ties in well with current definition of OOCSA but does include peers and those in relationships

#### A new normal

Online sexual harm appears common for children and young people. According to NSPCC (2020), there have been instances of children not using social media before the pandemic, using it for the first time and in some cases being immediately targeted by offenders. While taking a broader perspective on online sexual harm than used in the current paper, 9% of secondary school survey participants said they had learned about online sexual harm from personal experience (Beckett et al., 2019). Participants focused on sexual images, with females describing the “normality” of unsolicited explicit sexual images receipt, requests, and/or coercive messages to send such images to others (from peers, acquaintances, and strangers). With reference to harm, these participants intimated that such experiences could lead to desensitization, with incidents becoming part of everyday life rather than something harmful that required action.

Far less ambiguous for the children and young people in Beckett et al. (2019) study was that online sexual advances made by adults were more readily acknowledged as harmful by secondary school students. Participants felt that they had received more input on stereotypical “stranger danger” but, even here they acknowledged that the online environment made them more vulnerable to predators. This was reiterated by participants in Hamilton-Giachritsis et al. (2017, 2020a); some participants indicated that the online environment lowered inhibitions, creating a false sense of safety that contributed to hiding abusive dynamics and threats.

#### OOCSA grooming processes

Offenders across the studies display a wide range of grooming behaviors that monopolize on young people's vulnerability, sexual curiosity or inexperience. The NSPCC (2020) describes how perpetrators exploited young people's sense of isolation during COVID-related national lockdowns. Some children and young people spoke about being offered money in exchange for online sexual activity and that their families were under financial pressure during the pandemic. Manipulation of vulnerability and sexual curiosity was further demonstrated in Joleby et al. (2020) theme “*from thrilling to abusive.”* Young people describe a wide range of offender manipulation at the start of the abuse. This ranges from flattery that made them feel excited and gave them a self-esteem boost, right through to outright threats [also reported in NSPCC (2020) and Quayle et al. (2012)].

Threats were a key feature in Joleby et al. (2021) analysis of court documents. They found descriptions of children's experiences to fall into three themes; threatening situations, fear that someone would find out (through blackmail or IIOC reaching the public) and feeling they had no choice. The threat of IIOC was a key mechanism used by some offenders to maintain the victim's compliance with their actions. Wolak et al. (2018) found that perpetrators committing sextortion against minors (compared to adults) were more likely to pressure minors into producing IIOC, demand additional IIOC, threaten victims for more than 6 months and urge them to harm themselves. Joleby et al. (2020), Hamilton-Giachritsis et al. (2017, 2020a) and NSPCC (2020) also reveal anecdotal evidence of CSA offenders threatening suicide as another way to control child victims.

#### Offense related harm mechanisms

Two main offense-related characteristics were identified that could moderate harm outcomes; reach and IIOC-based offending including related threats.

Hanson (2017) argues that reach can moderate harm for online victims in a way similar to duration of abuse and use of force moderates harm for offline victims. Hamilton-Giachritsis et al. (2017, 2020a) highlight the role of technology in providing additional routes through which young people can be abused and controlled. NSPCC (2020) also observed perpetrators communicating with children in a variety of ways during the pandemic (multiple devices/platforms). In Hamilton-Giachritsis et al. (2017, 2020a) features of reach, such as night time access, led to victim immersion, fatigue, and concentration difficulties.

IIOC-based offending, including threats to share IIOC, moderates OOCSA harm in a similar way to that threats moderate harm for offline victims (Hanson, 2017). For OOCSA victims, self-blame is a key mechanism. Hamilton-Giachritsis et al. (2017, 2020a) found IIOC was linked to negative emotions, anxiety and self-blame (though not experienced by all young people) and that self-blame was related to complying with offenders' requests. Some children would comply with abuse rather than risk images of their abuse being seen by others (NSPCC, 2020), thus complicating the negative view they developed about themselves. IIOC shared with family members compounded feelings of embarrassment and shame (Whittle et al., 2013). For some, the fact that images might be used in the commission of future offenses, or used by other offenders for their own sexual gratification was even more impactful than them being seen by legal professionals (Hamilton-Giachritsis et al., 2017).

The findings from studies demonstrated that the permanence and reach of images can impact on day to day normal functioning (some interviewees described fear of leaving the house, fear of men) and lead to a sense of hopelessness about the future (career ambitions thwarted because of fear that images would resurface (Joleby et al., 2020). These negative impacts were also identified in a recent analysis of calls to ChildLine and counseling sessions following the first stay-at-home order (NSPCC, 2020).

#### Social and psychological contexts

Two contextual features were identified that could moderate harm outcomes; prior vulnerability and family support.

Four young people in Joleby et al. (2020) sample described their childhood before abuse as positive and safe, whereas three mentioned previous psychological problems. All were negatively affected by the online abuse, however, the three who had experienced prior problems described that the online abuse made “*everything collapse”* and they felt suicidal as a result. Whittle et al. (2013) confirm this qualitative assessment. They found that the two victims who self-harmed following abuse both had multiple long-term risk factors. In Whittle et al. (2013), irrespective of on or offline CSA, the impact of sexual abuse appeared to correlate most with young people's level of vulnerability prior to grooming onset. Those experiencing multiple long-term risk factors suffered greater negative impacts after grooming and abuse.

Supportive family relationships, in general and following CSA disclosure, play a crucial role in shaping long-term impacts (Hanson, 2017). In Whittle et al. (2013) individuals experiencing the greatest negative impact after grooming and abuse were more likely to experience unsupportive and negative responses from their families. However, these individuals also had more long-term risk factors. Hamilton-Giachritsis et al. (2017) also identified family support as a potential protective factor for victims following abuse. Family support was related to higher self-esteem and adaptive coping mechanisms. Lower levels of family support were associated with increased depression, anxiety, PTSD and shame. Quayle et al. (2012) also document a range of parental reactions, with negative impacts emerging when parents struggle to understand online CSA and in particular, their child's “participation” in the abuse.

#### Legal processes affecting harm

A focus on harm has revealed aspects of victim experience that help explain why some children do not recognize nor want to disclose their abuse. The privacy of the internet seems to play a considerable role in victims' ability to recognize or disclose abuse. In Joleby et al. (2020) some victims knew what they were doing “was wrong” but felt it was taking place privately, whilst others did not know they were being deceived by an adult offender until the police contacted the victim. As such, it was the detection process that triggered the internalization of them being a victim of online CSA. Detection processes, through police investigation and subsequent court proceedings, exacerbate feelings of self-blame and shame for some victims, contributing harm.

Interactions with the criminal justice system appear to play a role in determining impacts for children (Quayle et al., 2012; Whittle et al., 2013). In general CSA contexts, when children are interviewed multiple times, questioned harshly or perceive legal professionals to act unfairly or judge the children, this can have significant negative impacts on children (Hanson, 2017). In terms of OOCSA, young people intimate that the police investigation and court processes are particularly challenging as the extent of the abuse becomes public, including chat logs and images, leading to a feeling of shame among young people (Joleby et al., 2020). Additionally, Hamilton-Giachritsis et al. (2017, 2020a,b) highlight that poor practice with online CSA victims is not uncommon. Due to a lack of knowledge and understanding about online CSA professionals note that victims may be more likely to be 'blamed', seen as complicit in the abuse, the impact of the abuse might be minimized and there may be a failure to recognize it as abuse when it is online (Hamilton-Giachritsis et al., 2017, 2020b; amongst children and professionals). Joleby et al. (2021) found that vulnerability was mentioned less often in online CSA victim cases (12.3% of online CSA victim cases compared with 35.5% of offline CSA victim cases), which could indicate minimization if vulnerability and harmful outcomes are not afforded the same level of attention as in offline CSA cases.

Whittle et al. (2013) offer a slightly different view, as many children (across all victim vulnerability scenarios) provided many positive comments about police, counselors, social services and educational involvement. The length of police investigations was noted as a factor that could impact on wellbeing. Quicker resolutions were noted as helping to bring closure to victims so that they could move forward. Less positive experiences with police and social services cluster around vulnerable victims.

#### Comparisons with online CSA victims

The majority of included studies typically analyse accounts of CSA online victims with reference to offline CSA literature. Joleby et al. (2020) interview data confirms findings from their analysis of court documents (Joleby et al., 2021); that the range of psychological symptoms for victims of online CSA are similar to those reported among victims of offline CSA (psychological distress, self-harm and suicidal behavior, sleeping problems, impaired relationships, trust issues and difficulties at school [Whittle et al., 2013; also reiterated in NSPCC (2020)].

Two studies compared online and offline CSA. Considering a wide range of mental and physical health outcomes in a mixed-method study, Hamilton-Giachritsis et al. (2017, 2020a) report that Technology Assisted CSA (TA-CSA) is no less impactful than offline only CSA. Whilst their quantitative analysis was under-powered, their qualitative data pointed toward the same findings. Participants experiencing TA-CSA reported psychological difficulties similar to offline victims. Furthermore, practitioners did not perceive any less impacts of TA-CSA either (Hamilton-Giachritsis et al., 2017, 2020a,b). In their thematic analysis of eight interviews with CSA victims, Whittle et al. (2013) found no evidence to suggest that those who were also abused offline experienced a greater negative impact of abuse than those who were abused online only.

The severity of impact is apparent from the number of OOCSA victims who self-harm or suicide. Hanson (2017), for example, cites evidence from an informal scoping by CEOP in 2013 that found seven attempted suicides and seven completed suicides of young people in relation to online sexual blackmail. These victims were never physically touched and yet the impact on their lives was catastrophic, e.g., as illustrated in NSPCC (2020) - “*This guy knows everything about me... I'm terrified he's going to share these things with other despicable men on the internet. I can't deal with this anymore, I want to die”*(p. 5).

It is clear that victims experienced a range of harms, both in the long and short term. The majority of children in Joleby et al. (2021) described the online sexual act as either physically painful or distressing. Some interviewees did not realize the severity of their abuse until later (e.g., when contacted by police, Hamilton-Giachritsis et al., 2017, 2020a; Joleby et al., 2020). Others had been revictimised by multiple perpetrators and it was only later that they realized the extent to which they had been manipulated.

Wolak et al. (2018) report a number of outcomes for minors experiencing sextortion. These outcomes include loss of relationship with friends or family members (46%), seeing mental health or medical practitioners (29%), school related problems (14%), moving house (10%), financial costs (8%) and job related problems (8%). Only five percent of minors had none of these impacts. Interviewees who had personal experience of online sexual harm in Beckett et al. (2019) (albeit not clear whether from adults, peers, known or unknown) wanted others to understand the impact that online sexual harm can have, with “worst-case scenario” messages delivered in schools, as noted by one 16 year old female interviewee “*If they don't know, they won't know until it's too late. It can destroy your life*” (p. 8).

Further impacts on psychological health were noted in the literature. Joleby et al. (2020) evoke the narrative theme of ‘self-concept' and explain that young people now look at themselves differently after the abuse; the abuse affected their self-esteem, self-confidence, view of themselves as a “good person,” they experienced difficulties trusting people and had distorted views of their bodies and sex. The majority of studies acknowledge variation in long term outcomes (Quayle et al., 2012; Whittle et al., 2013; Hamilton-Giachritsis et al., 2017, 2020a,b; Hanson, 2017). Some children can come to terms with their experiences, contextualizing it as something that “happened in the past” (Quayle et al., 2012), whilst others struggle to maintain their psychological health and grow anxious about their futures. Factors influencing resilience and recovery appear to include the receipt of effective support services and sensitive response from family, friends and professionals following disclosure of CSA.

## Quality appraisal and discussion

This study assessed the quantity and quality of evidence exploring OOCSA related harms. The evidence base is developing. The majority of included studies would be best described as discovery focused and drawing upon unselected samples of the OOCSA victim population. The use of descriptive cross-sectional surveys (with limited case-control or longitudinal designs) and qualitative research are understandable given the recent research focus. It is apparent that researchers struggled to gain access to participants. This may relate to issues around self-blame and how victims view themselves along with research exploring their experiences. As such, the sample sizes described here are commendable. Power calculations were undertaken (Hamilton-Giachritsis et al., 2017) and reservations associated with sample sizes and underpowered studies are readily recognized by the study authors.

The quality, quantity and level of evidence for some findings (depression, anxiety, PTSD, suicide and self-harm) are promising but generalizability is low given the potential response bias that exists. Whittle et al. (2013) asked police and social service professionals to identify young people who might participate in their research. Hamilton-Giachritsis et al. (2017) recruited similar groups of young people through NSPCC services, ChildLine and the National Crime Agency (2020). Beckett et al. (2019) gained access to children who experienced online sexual harm through specialist support centers. Joleby et al. (2020) approach was broader, distributing recruitment flyers through lawyers, youth and psychology settings, social media and celebrities. Only Wolak et al. (2018) attempted to gain a community sample through recruitment on Facebook, but their study was limited to sextortion. Response bias may be apparent in police officers, treatment providers and lawyers identifying children who can talk about their abuse. Self-selected young people may more readily recognize their online experiences as harmful and/or have experienced more adverse outcomes. Study authors acknowledged bias but did not explicitly address it. Arguably, the field is not, as yet, developed enough and further work is needed to understand any potential differences between respondents and non-respondents. This is critical as non-respondents may feel they have not been harmed. However, there may also be those who experience such high levels of shame and self-blame that they do not wish to participate.

The second aim was to develop an understanding of online specific aspects of criminal behavior that contribute to harm. This might influence suspect or offense prioritization. Joleby et al. (2020) evoke the narrative theme of self-concept as a mediator between CSA and its negative outcomes for psychological health. They argue that, while each person's experience is unique, OOCSA can impact psychological suffering following the same patterns of offline CSA. Following this systematic review, it could be argued that the additional complications emerging from online abuse, reach, shame, self-blame and permanence of images, appear to be key factors that impact self-concept, as they affect how the young person views themselves, their futures and how they think they are viewed by others. Further harm mediators include prior vulnerability and poor social support. Such mediators are identified within the online and offline abuse literature (e.g., Fisher et al., 2017; May-Chahal et al., 2018; Jonsson et al., 2019).

A focus on harm has thrown up aspects of victim's experiences that are reminiscent of the literature on the cost of reporting and non-reporting among victims of sexual violence (see Walsh, 2012; Walsh and Bruce, 2014). Some victims report experiencing more severe PTSD due to legal participation. OOCSA can complicate victims' experiences and prevent them from wanting to cooperate with the police. Young people with multiple long-term risk factors may require considerable support from police and partner agencies to build rapport and gain victim cooperation. Police and social service failures to recognize the significant risk of harm, to acknowledge complicating factors emerging from the online offending environment, and respond to children in a sensitive way could contribute to harm outcomes as well as limit investigations. As such, it is critical to expand current Child Sexual Exploitation training and interventions to consider these online dynamics.

In terms of limitations, filtering criteria was used to identify relevant studies due to time pressures. However, efforts were taken to follow up included studies through the use of Social Science Citation Index. As it is likely that the limited number of studies published in this area cited each other, we can be reasonably confident that all studies were identified.

With reference to economic modeling, it is clear that some OOCSA victims experience adverse psychological outcomes requiring intervention in the same way that offline CSA victims do. What is less clear from this review is the proportion of victims that would need such a level of intervention. There is a clear continuum of harm here. At the one end, inappropriate sexual approaches have become the “new normal” for young people and the majority of individuals are not harmed. At the other end, OOCSA and offense-related harm mechanisms can lead to adverse psychological outcomes and suicide attempts. Whilst this review has not considered the number of victims typically harmed by an OOCSA offender it is unlikely that all children will be harmed to the extent experienced by some of the victims captured within this scoping review. Effort must be taken to avoid overestimating victim harm costs in subsequent economic analyses.

## Study 2: economic costs associated with OOCSA offenses

In the absence of OOCSA prevalence figures a decision was made to estimate prevalence from “online groomers” that fit with the working definition of OOCSA. Prevalence, and the costs outlined below, are applied to two population estimates.

### Calculating the prevalence of OOCSA offenses in the UK

#### Primary police data

UK police forces were asked to submit 16 closed cases of males, aged 18 years and over, with a conviction for online IIOC offenses (2014–2019) as part of a larger project to validate the existing KIRAT tool (Long et al., 2016). Given the lack of official data capture for OOCSA offenders, this data set acts as a sensible proxy. OOCSA offenders might be anticipated to come to notice through IIOC offending or to have committed IIOC offenses but come to notice through other means. The final data set included 486 cases. Around 43% of the sample engaged in online grooming (*n* = 206). Of these offenders six were identified by the first and fourth authors as fitting within the OOCSA definition. This represents a prevalence of 2.9% of online groomers being OOCSA offenders.

Population surveys were consulted to help verify this prevalence. Drawing upon Bergen et al. (2015) and Schultz et al. (2016), Wager et al. (2018) conclude that the proportion of adults holding sexualised conversations with a child is “unlikely” to be “below the lowest estimate of 1 in 10 adults” (Wager et al., 2018; IICSA, 2020). This holds for online sexual solicitation of adolescents (1 in 10) and children (1 in 100) from general website respondents (Schultz et al., 2016). Schultz et al. (2016) found almost a third of those who solicited minors reported making contact with more than 20 individual minors, 57% had interactions that lasted several days. Nearly half (47.5%) reported a sexual outcome, including 3.5% that had engaged in cybersex with an adolescent within the past year (1.1% with a child). The figure of 2.9% from police data fits within these values. As such, we can be reasonably confident that we are not overestimating OOCSA prevalence.

In the following section, the 2.9% prevalence of OOCSA is applied to two measures of online groomers. First, ‘sexual communication with a child' offenses recorded by police forces in England and Wales. These figures are also adapted to estimate undetected offenses. Second, the number of OOCSA offenders in England and Wales is estimated from Schultz et al. (2016) sexual solicitation prevalence estimates.

Economic costs are estimated across 43 police forces in England and Wales. To avoid overestimating harm, it is assumed that OOCSA offenders have harmed only one victim. This seems reasonable given that in Schultz et al. (2016) 5% reported interacting with more than 20 different minors in the past year. In Joleby et al. (2021) study 98 children were abused by 39 offenders.

#### Incidents across England and wales (43 forces)

Using Freedom of Information requests the NSPCC (2021) identified 5,441 Sexual Communication with a Child offenses recorded in England and Wales (42 Forces) for the year ending March 2021. This amounts to 130 offenses per force. If we scale up by adding an additional 130 (43 Forces) in England and Wales (*n* = 5,571) and apply 2.9% to this population there are an estimated 162 offenders recorded OOCSA offenses in England and Wales. We assume there are 162 unique offenders.

CSA is one of the most under-reported crimes. As study 1 demonstrates, victims of OOCSA may experience barriers to reporting given the complicated mixture of shame and self-blame they experience. Given that only 12% of CSA is estimated to come to the attention of statutory authorities (The Children's Commissioner for England, 2015) an attempt is made to quantify undetected offenses. If we take official figures as representing 12% of reported crimes, 162 offenses would increase to 1,350 national offenses. We assume unique offenders.

#### Prevalence of male and female online OOCSA offenders (43 forces)

Drawing upon Schultz et al. (2016) an assumption is made that 10% of the population are engaged in online sexual solicitation of adolescents; comprising 7% of males and 3% of females. Schultz et al. (2016) respondents were 18 to 80 years. However, the average age of online sexual solicitation offenders against adolescents was 24.5 years (SD = 7). So as to avoid overestimating the number of those engaged in online sexual solicitation we estimate 10% of the population within three standard deviations of the mean age (accounting for 88.89% of the set with non-normal data using Chebyshev's theorem). This would include individuals aged 18–46 years of age. This is likely to be a younger age range than observed in reality but avoids overestimation. Applying 7% male, 3% female and 2.9% OOCSA offending to population estimates of males and females in England and Wales in 2019 provides an estimate of 22,505 males and 9,522 females engaging in sexual solicitation of an adolescent and demonstrating OOCSA offending.

We could apply a further 1% of the population to online sexual solicitation of children, however among respondents from general websites in Schultz et al. (2016) the observed 1% was an aggregated age category (any child solicitation, not necessarily exclusive) and we do not wish to double count. They report 0.2% exclusive sexual solicitation of a child among general websites. We use this figure and estimate 0.14% of males and 0.06% of females. Taking three standard deviations, the average age of online sexual solicitation offenders against children is 28.5 years (SD = 9.9), which means applying prevalence estimates to individuals between 18 and 58. Applying 0.14% male, 0.06% female and 2.9% online OOCSA offending to population estimates of males and females in England and Wales in 2019 provides an estimate of 645 males and 276 females engaging in sexual solicitation of children and demonstrating online OOCSA offending.

In this estimate, the total population of OOCSA offenders in England and Wales = 32,948

### Calculating economic costs

A summary of measures used to estimate costs in anticipation of CSA, consequence of CSA and in response to CSA is provided in Table 3. In addition, how costs were adjusted to reflect OOCSA contexts.

**Table 3:** Summary economic model including adjustments made to account for OOCSA contexts.

	**Method**	**Financial or non-financial**	**Borne by**	**Radakin et al. (2021)**	**Adjustment for current study**
**Anticipation of Crime**					
Educational prevention programmes	Top down	Financial	Voluntary sector Government	Survey to offender focussed charities, rape crisis centers and other charities – typical annual spend on school programmes, youth engagement, targeted support for those at risk of CSA National Crime Agency spend on educational programmes	Focus on risk of child sexual abuse, assume spend applies to both contact CSA and OOCSA prevention (e.g., safety online) Radakin et al. (2021) prevalence increased to include OOCSA estimates Acknowledge spend applies to contact CSA and OOCSA
Offender prevention programmes	Top down	Financial and non-financial	Voluntary sector	Survey to charities that offer support to offenders – spending on counseling and support (financial) and number of volunteer hours (non-financial)	Adjustment as above Adjustment as above
CSA training costs	Top down	Financial Non-financial	Voluntary sector Government	Survey to offender focussed charities, rape crisis centers and other charities – spending on programmes delivered to adults, training professionals. Cost of contact CSA training by the police (£928,995)	Adjustment as above Costs related to contact CSA offending, removed from OOCSA calculations
**Costs as a Consequence**					
Physical and emotional harm	Bottom up	Non-financial	Victim	Likelihood of experiencing physical and emotional harms due to rape and or sexual assault, quality-adjusted life loss associated with physical and emotional harm, duration and frequency of physical and emotional harm, value of a life year, proportion of victims that experience rape only, rape and sexual assault and sexual assault only	Rape and physical injuries removed from consideration. Emotional costs associated with sexual assault retained for OOCSA calculation
Healthcare cost	Bottom up	Financial	Government	Likelihood of sustaining an injury, cost to NHS of treating physical and emotional injuries.	Costs of treating emotional injury associated with sexual assault retained for OOCSA calculation.
Lost output	Bottom up	Non-financial	Victim	Estimating difference between unemployment rates of CSA victims and non-victims.	Focus on CSA and not specifically contact CSA so assume increase rates of unemployment apply to OOCSA victims
Victim services	Top down	Financial and non-financial	Voluntary sector	Total expenditure on victim services (financial) (£24,143,825) and number of volunteer hours (non-financial) (£1.1 million)	Focus on risk of child sexual abuse, assume spend applies to both contact CSA and OOCSA prevention (e.g., safety online) Radakin et al. (2021) prevalence increased to include OOCSA estimates Adjustment as above
**Costs in response**					
Police costs	Top down	Non-financial	Government	Cost of an individual investigation into contact CSA is multiplied by number of investigations and divided by prevalence	Total police spend for rape and other sexual offenses divided by prevalence [contact CSA from Radakin et al. (2021), and OOCSA prevalence]
Court costs	Top down	Financial and non-financial	Government	Much of trial cost is based around costs for average offenders or general sexual offenses rather than contact CSA offenders.	Sexual Communication Offenses are an either way offense so could be heard in Crown Court or Magistrates Court, though culpability (e.g., use of threats, soliciting images) and victim harm are taken into account when determining offense category and potential sentence length (Sentencing council, 2023)^a^. Assume these features may have some bearing on whether case more likely brought to Crown Court
				Crown Prosecution Service costs for prosecuting any crime at Crown Court level (financial) Crown Court costs including average hours for sexual offense trials (financial) Legal aid costs for average Crown Court case (financial) Jury service costs based on adult sexual offenses (non-financial)	Not specific to CSA or contact CSA – no adjustment made Average hours in Crown Court for adult sexual offenses used as proxy. Given amount of digital evidence may not be unreasonable to assume OOCSA cases likely to be as long as adult sexual offenses – no adjustment made Like Radakin et al. (2021) assume all CSA offenders receive legal aid Medium number of hours for adult sexual offenses used as proxy – adjustment made **Total** Prosecutions are roughly 6% of reported contact CSA across years [2018 = 3500 and 2019 = 3,768 as reported in Radakin et al. (2021) Assume 6% cases prosecuted
Prison costs	Top down	Financial	Government	Cost of custody for those sentenced for contact CSA offenses within a given year (multiple annual cost per prisoner by average sentence length for CSA offenders in years and number of offenders sentenced to custodial sentences in a given year) divided by prevalence estimate Cost of non-custodial sentences calculated using estimates for the average offender divided by prevalence estimate	Roughly 2.6% of contact CSA offenses resulted in custodial sentence in figures used by Radakin et al. (2021) Assume 2.6% cases custodial sentence Tariff for sexual communication offenses up to 2 years – assume one year at annual cost of £41,135 provided by Radakin et al. (2021) Roughly 1.1% of contact CSA offenses resulted in non-custodial sentences in figures used by Radakin et al. (2021) Assume 1.1% cases non-custodial sentence Estimated cost provided by Radakin et al. (2021) £4780
Safeguarding costs	Top down	Financial		Number of children in each safeguarding category due to contact CSA multiplied by the average length of time a child spent in the safeguarding category/ multiplied by cost of a year in each safeguarding category/ divided by prevalence estimate	Cannot know proportion of 29% children (Radakin et al., 2021) that would feasibly be estimated to be OOCSA victims. Safeguarding costs removed from consideration

^a^Sentencing council - https://www.sentencingcouncil.org.uk/offences/crown-court/item/sexual-communication-with-a-child/ (accessed December 11, 2023).

Radakin et al. (2021) provide an estimate of the financial and non-financial costs related to all children who experienced contact CSA (new or continuing cases) in England and Wales in the year ending 31st March 2019. “Financial” costs indicate direct cash and budgets associated with CSA. “Non-financial” costs refer to all other costs, including “notional” values which estimate harm in monetary terms. They provide a range of “bottom-up” and “top-down” costs. Radakin et al. (2021) estimated bottom-up costs on a per victim basis. Subtractions are considered here where it might be feasible to suggest that OOCSA victims would not incur such a cost (e.g., physical injuries). “Top-down” costs consider wider budgets for services supporting victims divided by the prevalence of contact CSA victims. Where these services might equally apply to both contact CSA and OOCSA the divisor is amended to include estimates of OOCSA so that the total prevalence includes both contact CSA (113,114 Radakin et al., 2021) and OOCSA victims. OOCSA unit costs and total costs are calculated using the same methodology as Radakin et al. (2021). Given the longitudinal nature of some costs these are not annual costs.

## Study 2 results

Table 4 summarizes unit and total costs across the three prevalence estimates. Table 5 establishes whether that cost is borne by the government, voluntary services or victims themselves and whether the cost is financial or non-financial. Over 75% of estimated costs represent a non-financial burden experienced by victims. The government (through healthcare, policing, courts, prisons etc.) pays the majority of the remaining costs. This is mainly non-financial, where existing services could be used elsewhere if OOCSA offenses stopped.

**Table 4:** Estimated unit and total costs across three OOCSA prevalence estimates.

	**Radakin et al. (2021) *n =* 113,114**	**Lower bound *n =* 162 OOCSA**	**Middle bound *n =* 1,350 OOCSA**	**Upper bound *n =* 32,948 OOCSA**
**Educational prevention programmes**				
Surveys	£25	£20	£20	£15
National crime agency	£5	£5	£5	£5
**Offender prevention programmes**				
Surveys	£20	£20	£20	£15
Volunteer hours	£0	£0	£0	£0
**CSA training costs**				
Survey	£10	£10	£10	£10
Police CSA training	£10			
Physical and emotional harm	£45,985	£23,190	£23,190	£23,190
Healthcare cost	£1,545	£380	£380	£380
Lost output		£11,550	£11,550	£11,550
**Victim services**				
Total expenditure	£215	£215	£210	£165
Volunteer hours	£10	£10	£10	£10
Police costs	£8,625	£8,615	£8,525	£6,680
Court costs	£365	£725	£95	£5
Prison costs	£3,085	£1,075	£125	£5
Safeguarding costs	£17,800			
Total unit cost	£89,240	£45,815	£44,140	£42,030
Unit cost^*^prevalence		£7,422,030	£59,589,000	£1,384,804,440

**Table 5:** Unit costs, total costs and % total costs for government, voluntary services, and victims.

	**Government**						**Voluntary**						**Victims**		
	**Financial**			**Non-financial**			**Financial**			**Non-financial**			**Non-financial**		
	**Low**	**Mid**	**High**	**Low**	**Mid**	**High**	**Low**	**Mid**	**High**	**Low**	**Mid**	**High**	**Low**	**Mid**	**High**
Unit cost	£2,135	£590	£395	£8,665	£8,540	£6,680	£265	£260	£205	£10	£10	£10	£34,740	£34,740	£34,740
Total cost	£345,870	£796,500	£13 million	£1.4 million	£11.5 million	£220.1 million	£42,930	£351,000	£6.8 million	£1,620	£13,500	£329,480	£5.6 million	£46.9 million	£1.1 billion
% Total cost	5%	1%	1%	19%	19%	16%	1%	1%	1%	0% (0.02)	0% (0.02)	0% (0.02)	76%	79%	83%

The estimated lifetime cost associated with one victim per 162 OOCSA offenders (official police reports) is £45,815. The total lifetime cost is £7.4 million.

The estimated lifetime cost associated with one victim per 1350 OOCSA offenders (including undetected offenders) is £44,140. The total lifetime cost is £59.6 million.

The estimated lifetime cost associated with one victim per 32,948 (self-report survey) is £42,030. The total lifetime cost is £1.4 billion.

Police costs range from £1.4 million to £220.1 million. These are costs that could be diverted to other areas of police business.

## Discussion

Study 2 presents three ways in which the socio-economic burden potentially contributed by OOCSA offenders can be estimated. To be clear, these estimates refer to offending that is initiated and takes place online only. It does not include offending that is initiated online and takes place offline. Given NCA (2023) recent estimates of 680,000 to 830,000 UK based adult offenders posing an online and offline threat it is likely that the number of online only offenders is larger but the present research provides an evidence-led starting point in discussions around this specific online sub-population. This work demonstrates the potential scale of the problem in England and Wales. Relatedly, an economic framework is provided that could help develop strategy and understanding around the amount of investment needed for an effective policing response.

In the first analysis, we consider the amount of adults across England and Wales who have committed an offense of “sexual communication with a child” and who might contribute OOCSA related harm to at least one victim. The estimated lifetime economic burden attributable to these offenders is around £7.4 million. The second analysis takes official figures accounting for only 12% of the real number of offenses and calculates the number of undetected offenders. The estimated lifetime economic burden increases to £59.6 million. To provide an evidence-based upper bound prevalence estimate we draw on Schultz et al. (2016) survey of self-reported child sexual solicitation. The estimated lifetime economic burden attributable to these offenders is £1.4 billion. Whilst this is likely an overestimate we have to accept that this is a relatively new form of offending. The number of offenses may increase if appropriate action is not taken to address and prioritize the real harm caused to victims. Official reports provide only a snapshot, and we might expect that online crimes will continue to increase.

Most costs are non-financial costs borne by victims. Whilst it might seem cold or reductive to think of harm in this way it helps to make tangible the disadvantages experienced by victims in the short and long term. Some might question the use of emotional harm costs from contact victims as that might overestimate the cost of emotional harm for OOCSA victims. However, based on Norris and Kaniasty (1994) work on fear of crime and Dolan et al. (2005) work on victims of violent crime, Radakin et al. (2021) calculation assumes that only 25% victims will experience fear for 1.25 years, 9% will suffer depression for 1 year, and 20% will experience anxiety/panic attacks over 3 years. Secondary health problems such as drug abuse, alcohol abuse and eating disorders are not included. We cannot know the extent to which these estimates overestimate emotional harm, as data is not available for OOCSA victims. However, findings from study 1 suggest that these conservative estimates might conceivably apply to OOCSA. Likewise, we do not believe it unreasonable to apply cost estimates based on the difference between unemployment rates of contact CSA victims and non-victims to OOCSA victims. Study 1 revealed preliminary evidence around education and employment for some victims, especially in the context of threats, blackmail and the permanence of images. It may be that employment disadvantages are even more pronounced for image-related offending whether offending takes place online or in person.

After victims, the majority of the remaining cost is borne by police forces in terms of non-financial costs that could be diverted elsewhere if OOCSA offending stopped. The police costs for OOCSA offenses were calculated by dividing the total police spend for sexual offenses by the prevalence of contact CSA and OOCSA. This is a sensible strategy and is likely to be an underestimate of police costs given the complexity of police investigations particularly those involving digital evidence. Christie (2018) for example, used a bottom-up approach to estimate police costs associated with victim and perpetrator journeys through investigation and court processes and found CSA offenses with an online element cost £18,499. This is far higher than the top-down approach adapted from Radakin et al. (2021) and utilized here.

It is important to consider how these findings should be used. First, it is important to reiterate that these are not annual costs. These are estimated costs incurred over the lifetime of victims abused during a year. Police data looked at “sexual communication with a child” offenses over a year (year ending March, 2021). Schultz et al. (2016) asked respondents whether they had engaged in behaviors in the past 12 months. A victim will continue to experience impacts over a long period of time. If researchers treated these as annual costs then they would double count impact in subsequent years. Only new victims generate new costs, not historical victims.

To help avoid such costs and reduce offending police efforts might be targeted toward those offenses that are most likely to contribute to harm as outlined in study 1. Drawing upon findings from study 1 and study 2, police forces should raise awareness about OOCSA prevalence and victim harm. This approach might also be extended to parents and care givers so that they can more effectively support their child. The present work has also demonstrated that criminal justice responses to victims need to explicitly deal with shame and self-blame. This accords with other work such as Operation Soteria (see Stanko, 2022) that recognizes the need for improvements in victim interventions more generally. Further empirical work has demonstrated adverse effects that can be experienced by child victims during police interviews. For example, Lewy et al. (2015) found the use of non-supportive comments and failure to effectively address children's reluctance during CSA police interviews impacts on disclosures from children. Similarly, Kim et al. (2020) found that rapport-based police interviewing techniques are effective in improving interview yield from child CSA victims. Further investment in training and rapport-based approaches might help to alleviate the harms experienced by OOCSA victims. In terms of suspect or offense prioritization it is imperative that a detailed assessment is undertaken to understand victim vulnerability and harm. Arrest prioritization strategies might be adapted that escalate risk in cases involving multiple victims, image-based offending, image-based threats and vulnerable victims irrespective of contact offending risk.

There are several potential limitations to our approach. First, there may be problems with police data or estimates derived from police data. First, police forces were asked to submit 16 cases across 5 years. Not all forces sent 16 cases. There must be some reservations about the extent to which this sample reflects a true representation of IIOC and OOCSA offenders. However, there is a lot of variety within the data set and we verified the 2.9% estimate against self-reported sexual solicitation offenders who engaged in cybersex with minors. Second, Sexual Communication with a Child Offenses may be a questionable proxy for OOCSA offending. Due to the Home Office Counting Rules, the most serious offense in a case must be recorded by police (McGuire and Dowling, 2013). It is unclear from NSPCC reports whether data returned by forces in their FOI requests are the most serious offenses or whether offenders committed other offenses. There may be OOCSA aspects of other more serious offenses that are not captured in the present data. This means that prevalence may be underestimated here. Third, we conflate offenses with offenders and assume that police reports are made about distinct individuals. This may have led to overestimates of the number of unique offenders for both the lower bound and middle bound prevalence estimates. To offset this we assume that only one victim is harmed per offender. Further work would be useful to establish the number of repeat offenders in criminal samples along with the number of victims approached and harmed.

There may be additional limitations to Schultz et al. (2016). Their methodology involved recruiting participants from German, Finnish and Swedish websites. The majority of respondents soliciting minors were recruited from German websites. The proportion of German participants soliciting minors was significantly higher than Sweden and Finland combined. Schultz et al. (2016) discuss these findings in terms of legal deterrence and that legislation in Sweden and Finland may have affected disclosure by perpetrators. Various offenses related to online sexual solicitation are available under UK law and would likely affect research participation in similar ways to Sweden and Finland. It is difficult to know whether prevalence is similar to a UK sample. In Schultz et al. (2016) the majority of participants soliciting minors were from a small subsample recruited from pedophilia-related websites. They argue that these individuals pose a higher risk. As such we cannot be confident that 3.5% offenders from general websites engaged in cybersex as the data is not broken down in this way. Nonetheless, they do make clear there was no increased probability of achieving sexual outcomes for respondents from paedophila-related websites compared to general websites. Finally, the cost burden may be inflated by the number of female offenders estimated to pose a risk of OOCSA harm in this study. Some might argue that 30% is too high and it would be beneficial to gain police perspectives on this. Female sexual offenders represent around 4–5% of all sexual offenders (e.g., Sandler and Freeman, 2007; Almond et al., 2015). Female internet offenders may be rarer in the criminal justice system, due to difficulty detecting online offenses and low rates of detection of female offenses (Elliott and Ashfield, 2011). Schultz et al. (2016) use self-report survey methods, which are less susceptible to offender deception. Researchers should consider the proportion of male and female offenders in the UK. It is important for police forces and victims to be aware of the risks posed by offenders of both genders.

Whilst the cost estimates we present here are alarming, we believe they are useful as a starting point in the discussion around OOCSA offending. We have tried to identify conservative estimates so as not to overestimate the problem scope. First, we adapted the detailed report produced by the UK Home Office. Costs associated with physical injuries were removed and where it was too difficult to provide an evidence-led estimate (e.g., safeguarding) it was removed from cost considerations. Second, the estimates provided here explore adult offending costs only. It is likely that the costs arising from adolescents offenders are also considerable. Third, this type of offense appears to be more prevalent among younger offenders. To avoid overestimating prevalence using Schultz et al. (2016) we estimated offenders within three standard deviations of the average age of online sexual solicitation offenders. Finally, economic harm does not consider impact on family members (e.g., Quayle et al., 2012; Hanson, 2017).

There are several ways the present study could be developed. The OOCSA economic model could be explored further. Cost of OOCSA training by the police was not included in the present economic model. Court costs and prison costs were estimated from figures available in Radakin et al. (2021) but it would be useful to explore whether OOCSA offenses are heard in magistrates or crown court and what the typical sentence is when offenders are found guilty. Further work exploring victimization would also be beneficial. As study 1 demonstrated some victims do not experience significant psychological difficulties following sexual abuse (Hanson, 2017) and among those that do, some overcome this. We do not know from this analysis how many people are impacted by each offender operating online. We do not know how many victims experience the types of impact accounted for in our economic model. A more realistic assessment of victim numbers could drastically alter the estimates provided here. Further development is needed in establishing the vicarious costs associated with being a victim of an OOCSA related IIOC offense. In this research, image permanence is emphasized, but it is not considered in economic models. Evidence is emerging that highlights the additional impact of IIOC distribution on its victims. Many victims report that additional distress is caused by knowing that images of their abuse are circulated and viewed for sexual purposes (von Weiler et al., 2010) leading some authors to conclude that this constitutes revictimisation in real terms as victims experience exacerbated PTSD symptoms including flashbacks and panic attacks. This may be one area where the economic burden, in terms of long term health impacts on contact CSA and OOCSA victims needs to be considered in much more detail.

This research explored the scale of OOCSA offending problem, impact on victims and resulting implications for police response and suspect prioritization. It provides the first economic appraisal of OOCSA offenders in England and Wales. We anticipate that the costs presented here are conservative. And yet, they offer a starting point for further discussion about how such costs can be calculated and police efficacy evaluated. This research justifies police investment in this area. A number of evidence-based recommendations are provided that suggest what that strategy might look like.

## Data availability statement

The datasets presented in this article are not readily available because controlled and sensitive data (i.e., collected for purposes of KIRAT validation) is not available to anyone outside the research team. Requests to access the datasets should be directed to lalison@liverpool.ac.uk.

## Author contributions

SG: Conceptualization, Data curation, Formal analysis, Methodology, Writing—original draft. LA: Funding acquisition, Supervision, Writing—review & editing. MH: Data curation, Funding acquisition, Project administration, Resources, Writing—review & editing. RT: Data curation, Methodology, Writing—review & editing. HR: Data curation, Project administration, Resources, Writing—review & editing.
